# Nontuberculous Mycobacteria in Tap Water

**DOI:** 10.3201/eid1802.110455

**Published:** 2012-02

**Authors:** Eduardo Hernández-Garduño, Kevin Elwood

**Affiliations:** British Columbia Centre for Disease Control, Vancouver, British Columbia, Canada

**Keywords:** nontuberculous mycobacteria, Mycobacterium avium, tuberculosis and other mycobacteria, bacteria, tap water, boiling, risk factors, gastroesophageal reflux disease

**To the Editor:** A recently published study by Falkinham (*1*) showed that 17 (46%) of 37 households were contaminated with nontuberculous mycobacteria (NTM) of the same species as those found in patients with lung disease and that 7 (41%) of 17 had the same DNA fingerprint as the patient. One patient’s isolate from sputum matched the isolate found in the shower water. Therefore, the patient’s lung disease was likely acquired by inhalation of aerosols while showering. An isolate from another patient matched the isolate found in tap water. If the patient drank the contaminated water, *Mycobacteria* *avium* may have reached the lungs by aspiration because 26% of patients with NTM lung disease have been found to experience gastroesophageal reflux disease (GERD) (*2*). Even if none of these scenarios was present, however, NTM patient contamination of samples is still likely. Six of the 7 matching households had water heater temperatures <125°C, indicating a negative correlation between NTM growth and temperature. Most *M*. *avium* and *M*. *intracellulare* are killed in <5 seconds (*3*) when exposed to 70^o^C; thus, all NTM species would likely be killed a few seconds after water reached the boiling point.

In a recent study, we have shown that Canadian-born persons from ethnic groups from eastern and Southeast Asia were less likely to be colonized with *M*. *avium* complex than were other ethnic groups (*4*). We hypothesized that boiling water before consumption, a common practice in persons from Asia, may have partially protected them against pulmonary colonization. Another protective factor is the low prevalence of GERD in persons from Asia (<7%) (*5*), compared with 19.8% in white persons from Olmstead County, Minnesota, USA. Future studies like that of Falkinham are needed to determine routes of transmission. Factors to investigate in such studies include the ethnicity of participants and associated predisposing disorders, particularly GERD; culturing of gastric washings; handwashing frequency; and water consumption habits (whether drinking from the bottle, from the tap, or after boiling).
